# The Light-Regulated *SsMYB106* Transcription Factor Promotes Flavonoids in *Spatholobus suberectus*

**DOI:** 10.3390/ijms26115292

**Published:** 2025-05-30

**Authors:** Shuangshuang Qin, Ying Liang, Fan Wei, Guili Wei, Quan Lin, Xiaoying Chen

**Affiliations:** 1 Guangxi Key Laboratory of Medicinal Resources Protection and Genetic Improvement, National Center for Traditional Chinese Medicine (TCM) Inheritance and Innovation, Guangxi Botanical Garden of Medicinal Plants, Nanning 530023, China; qin_double@126.com (S.Q.); 198yingzi@163.com (Y.L.); fanwei_gx@163.com (F.W.); weigl1992@163.com (G.W.); 15607809304@163.com (Q.L.); 2 National Engineering Research Center for Southwest Endangered Medicinal Materials Resources Development, Guangxi Botanical Garden of Medicinal Plants, Nanning 530023, China

**Keywords:** flavonoid biosynthesis, catechins, anthocyanins, transient overexpression, stable overexpression

## Abstract

*Spatholobus suberectus* Dunn is rich in flavonoids, which were previously demonstrated to respond to changing light conditions. In this study, through weighted gene co-expression network analysis (WGCNA), *MYB* genes were screened as the potential regulator for these light-intensity-induced changes. One *MYB* gene, *SsMYB106*, was chosen for in-depth research. Analysis showed that *SsMYB106* was an R2R3-type MYB transcription factor, regulated by light, and involved in controlling the light responses of *S. suberectus*. *SsMYB106* was then transiently overexpressed in *S. suberectus* flowers and stably overexpressed in *Nicotiana benthamiana* Domin. *SsMYB106* overexpression promoted flavonoids (especially catechins) accumulation, and affected expressions of all the flavonoid biosynthetic pathway genes. In transient overexpression, *SsDFR1* was the only significantly decreased gene, while other 17 genes were significantly increased. In stable overexpression, nearly all genes were upregulated or at least unchanged. *SsMYB106* overexpression also might function in inhibiting anthocyanin and lignin biosynthesis. This study deepens our understanding of *SsMYBs* gene functions in regulating and enhancing flavonoids, and would be beneficial for designing high-valued *S. suberectus* in future.

## 1. Introduction

*Spatholobus suberectus* Dunn belongs to the genus *Spatholobus*, family Fabaceae. *S. suberectus* is a popular ornamental plant that brings auspiciousness and longevity in Chinese traditional culture. The dried vine stem of *S. suberectus*, called *Spatholobi caulis*, is a traditional Chinese herbal medicine. Historically, *S. suberectus* was universally used for the therapy of promoting hematopoiesis, and currently the utilization is broadening to the treatment of tumor, leukopenia, diabetes, osteoporosis, bacterial/viral infections, etc. [1,2]. *S. suberectus* is also edible and used for making wine, syrup, tea, and soup. For instance, it can be cooked with egg, chicken, duck, pork, and other herbal medicines (*Salvia miltiorrhiza* Bunge, *Forsythia suspensa* (Thunb.) Vahl, *Angelica sinensis* (Oliv.) Diels, *Ligusticum sinense* ‘*Chuanxiong*’, and so on) to make Chinese medicated diets.

The predominant bioactive components of *S. suberectus* are flavonoids. In *S. suberectus*, the main flavonoid biosynthetic pathway can be divided into the following three stages:

The first stage [3,4,5] converts phenylalanine to 4-coumarate CoA, including three enzymes: Phenylalanine ammonia-lyase (PAL), cinnamate-4-hydroxylase (C4H), and 4-coumarate CoA ligase (4CL). This stage is also called the general phenylalanine/phenylpropanoid pathway.

The second stage [3,6,7,8] converts 4-coumarate CoA to dihydroflavonols (dihydrokaempferol and dihydroquercetin), including four enzymes: chalcone synthase (CHS), chalcone isomerase (CHI), flavanone 3-hydroxylase (F3H), and flavonoid 3′-hydroxylase (F3′H). In this stage, CHS mediates the formation of naringenin chalcone from 4-coumarate CoA and directs the metabolic flux into the flavonoid pathway. CHI functions in the production of naringenin, a pivotal precursor for many flavonoids. F3H and F3′H catalyze the formation of dihydroflavonols. Additionally, chalcone reductase (CHR) catalyzing the production of isoliquiritigenin from naringenin chalcone is also classified into this stage.

The third stage [7,8,9,10] converts dihydroflavonols to leucoanthocyanidins by dihydroflavonol-4-reductase (DFR), and then diverges into two branches: One branch pathway is catechin biosynthesis, converting leucoanthocyanidins to catechin/gallocatechin by leucoanthocyanidin reductase (LAR), or converting leucoanthocyanidins to epicatechin/epigallocatechin by anthocyanidin synthase (ANS) and anthocyanidin reductase (ANR). Catechins are the most abundant flavonoids in *S. suberectus*. The other branch pathway is anthocyanin biosynthesis, converting colorless leucoanthocyanidins to colored anthocyanidins (pelargonidin, orange-red; cyanidin, red-purple) by ANS. Then the unstable anthocyanidins are glycosylated or acylated to form stabilized anthocyanins, and transported into vacuole for storage.

The regulatory network of flavonoid biosynthesis is not well elucidated in *S. suberectus* yet. It is unknown what types of transcription factors (TFs) regulate the different flavonoid biosynthesis branches and how they act on targeted genes. Our previous study demonstrated that MYB (*v*-*myb* avian myeloblastosis viral oncogene homolog) TFs, particularly R2R3-MYBs, are involved in flavonoid biosynthesis regulation of *S. suberectus* [7]. From the work of other researchers, although there are species-specific regulatory mechanisms, normally, MYB TFs transcriptionally regulate flavonoid biosynthesis through three ways: The first is physically binding to the promoter of target genes, thereby activating or suppressing their expressions and directly regulating flavonoid biosynthesis [11,12,13,14]; the second is working through the MYB-bHLH-WD40 (MBW) ternary protein complex, thereby coordinately controlling flavonoid biosynthesis [15,16,17]; the third is interacting with other MBWs/TFs, thereby directly or indirectly modulating flavonoid biosynthesis [5,18]. In *S. suberectus*, we previously identified 272 *SsMYB* genes and discovered that 22 of them were potentially associated with flavonoid biosynthesis [19], but the exact molecular mechanisms of how MYBs regulate flavonoid biosynthesis in *S. suberectus* were not well illustrated.

Flavonoids (especially catechins) contents and associated biosynthetic gene expressions were previously proved to change with light conditions [8]. The objective of this study is to search for the underlying regulator genes of these light-intensity-induced changes and explore gene functions in regulating flavonoid biosynthesis. For this aim, the weighted gene co-expression network analysis (WGCNA) was used for data mining. The chosen *SsMYB106* gene characterization and its correlations with flavonoids and biosynthetic genes were discovered. Then, *SsMYB106* was transformed into *S. suberectus* transiently and *Nicotiana benthamiana* Domin stably to explore gene functions. Finally, a working model was proposed and future work was suggested. Our research provided knowledge of *SsMYBs* gene functions in regulating flavonoid biosynthesis, and could be utilized for the molecular breeding of *S. suberectus* and synthetic biology of plant flavonoids.

## 2. Results

### 2.1. WGCNA Revealed MYB TFs Were Highly Associated with Light-Induced Flavonoid Content Changes in S. suberectus

**Figure 1:**
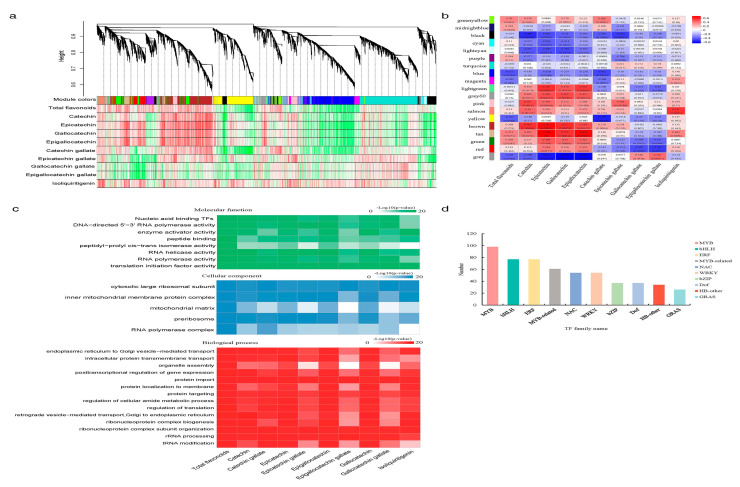
Discovery of *MYB* genes through WGCNA. (**a**) Hierarchical cluster tree. Each branch in the gene dendrogram corresponds to a color-coded module. Color rows underneath denote the assigned co-expression modules and heatmap plot of traits. (**b**) Module-trait correlation heatmap. Each row represents a color-coded module and each column represents a specific flavonoid compound (or total flavonoids). Numbers in each cell represent the module-trait Pearson correlation coefficient and the corresponding *p*-value (in bracket). Red/blue color represents positive/negative correlation, and the deeper color represents the stronger correlation. (**c**) Gene Oncology (GO) analysis. Positive correlated genes in the module-trait correlation heatmap were functionally assigned to molecular function, cellular component, and biological process. (**d**) The top ten WGCNA screened transcription factors (TFs). MYB, *v*-*myb* avian myeloblastosis viral oncogene homolog; bHLH, basic helix-loop-helix; ERF, ethylene response factor; MYB-related, contains an intact or partial MYB repeat (1R-MYBs); NAC, contains three conserved domains—petunia no apical meristem (NAM), ATAF1/2, and cup-shaped cotyledon (CUC); WRKY, contains a conserved WRKY (WRKYGQK) domain at the N-terminal end; bZIP, basic leucine zipper; Dof, DNA binding with one finger; HB-other, homeobox (HB) contains a conserved homeodomain (HD) domain; GRAS, GIBBERELLIN-ACID INSENSITIVE (GAI), REPRESSOR of GA1 (RGA), and SCARECROW (SCR), namely, GAI-RGA- and -SCR (GRAS) protein, contains a C-terminal GRAS domain with a highly variable N-terminal region.

Our previous study revealed that flavonoid contents and their biosynthetic pathway structural genes in *S. suberectus* were responsive to varied light intensities and durations (light treatments, including four attenuated light intensities (100%, 60%, 40%, and 10%) and three durations (30 d, 45 d, and 60 d)) [8]. To search for the regulatory genes related with these light-intensity-induced changes, flavonoids content data (total flavonoids, eight catechins, and isoliquiritigenin) and transcriptional RNA-seq data (33,525 genes in all) in the light treatments were used for WGCNA. A total of 19 color-coded modules were identified by hierarchical clustering (Figure 1a), and a module-trait correlation heatmap presented the positively (red) and negatively (blue) correlated ones (Figure 1b). Of all the 16 positive correlations, 12 modules, marked as “greenyellow”, “midnightblue”, “magenta”, “lightgreen”, “grey60”, “pink”, “salmon”, “brown”, “tan”, “green”, “red”, and “grey”, were significantly correlated with the flavonoids contents, while four modules, marked as “cyan”, “purple”, “turquoise”, and “blue”, were non-significant (Figure 1b). Next, the 16 positively correlated modules were all used for Gene Oncology (GO) enrichment analysis (Figure 1c). GO annotation describes a gene product’s molecular-level functions, the cellular locations where it performs functions, and the multiple biological processes in which it participates. Analysis of the GO molecular function terms indicated that TFs were highly positively correlated with flavonoids contents in *S. suberectus* (Figure 1c). Further analysis showed that MYB TFs were the most abundant of the top ten WGCNA screened TFs (Figure 1d). We chose an *MYB* gene on chromosome 6 (SsMYB_Chr6.1555), *SsMYB106*, to explore how it functioned in the light-intensity-induced flavonoid content variations in *S. suberectus*.

### 2.2. *SsMYB106* Was Characterized as a Light-Regulated R2R3-MYB TF in S. suberectus

**Figure 2:**
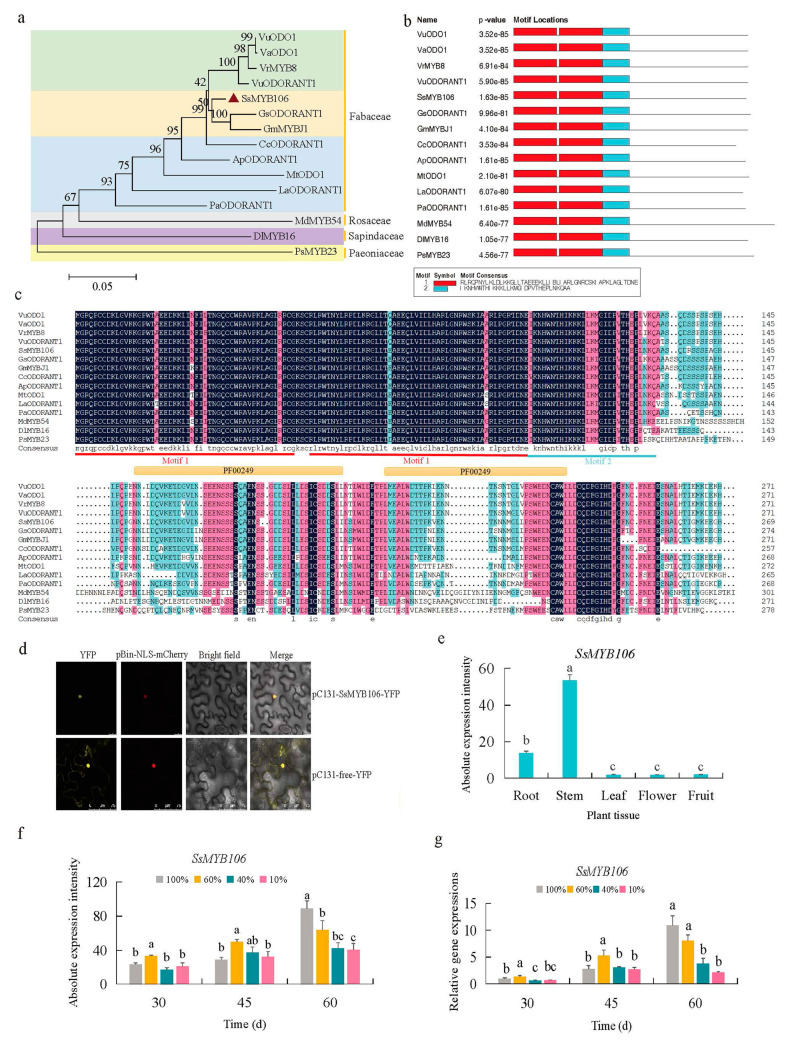
*SsMYB106* phylogenetic relationship, R2R3-MYB domain, subcellular localization, and expression pattern. (**a**) Phylogenetic tree. The neighbor-joining tree was constructed with 1000 bootstraps in the MEGA11 package. The scale bar represents 0.05 amino acid substitutions per site. The number at the nodes indicates bootstrap value. The transcriptional factors’ GeneBank sequence accession numbers in NCBI were: VuODO1, SPEB01002064; VaODO1, CM003371; VrMYB8, JJMO01000123; VuODORANT1, NBOW01000003; *SsMYB106*, TKY55375; GsODORANT1, QZWG01000017; GmMYBJ1, ACUP04001638; CcODORANT1, AGCT01010269; ApODORANT1, QYUI01000146; MtODO1, CM001220; LaODORANT1, KV862056; PaODORANT1, SMJV01004691; MdMYB54, AUZ96353; DlMYB16, QCH41128; and PsMYB23, QIG55706. (**b**) Motif discovery. Motif 1 and Motif 2 were two R2R3-MYB motifs containing SANT/myb domains (e.g., PF00249) discovered by MEME (Version 5.5.2). (**c**) Multiple amino acid sequence alignment by DNAMAN (Version 6.0). The red and blue lines denote two MEME discovered motifs (Strat-end positions of Motifs: Motif 1, 11–50, 54–103; Motif 2, 104–134). The orange lines denote two InterPro (Pfam entry: PF00249) Myb-like DNA-binding domains (Strat-end positions of PF00249: 14–61, 71–111). (**d**) *SsMYB106* subcellular localization in lower epidermal cells of *N. benthamiana*. YFP, yellow fluorescent protein; pBin-NLS-mCherry, red fluorescent protein fused with nuclear localization signal; Bright field, the transmitted light image; Merge, the transmitted light image overlayed with YFP and mCherry fluorescence image. Scale bar, 75 μm. (**e**) *SsMYB106* tissue-specific gene expression in five tissues of eight-year-old cultivated *S. suberectus*, quantified by RNA-seq. (**f**,**g**) *SsMYB106* light-intensity-induced gene expression in stems of two-year-old cultivated *S. suberectus*, quantified by RNA-seq (**f**) and RT-qPCR (**g**). Lower case letters indicate statistically significant differences caused by light intensity (*p* < 0.05, a > b > c > d).

The phylogenetic analysis of the *SsMYB106* protein in *S. suberectus* and its homologous proteins in 14 other species (Figure 2a) showed that *SsMYB106* was clustered with wild soybean GsODORANT1 protein (*Glycine soja*, XP_028209493.1) and domesticated soybean GmMYBJ1 protein (*Glycine max*, NP_001241087.1). Moreover, *SsMYB106* was distantly related to MdMYB54 (*Malus pumila*, AUZ96353.1), DlMYB16 (*Dimocarpus longan*, QCH41128.1), and PsMYB23 (*Paeonia* × *suffruticosa*, QIG55706.1). These results indicated that *SsMYB106* might have functional similarities to the two most closely related proteins, and these results were consistent with *S. suberectus’* origin of Fabaceae ancestor.

The conserved domains of *SsMYB106* protein and the 14 homologous proteins were searched by MEME (Version 5.5.2) and InterPro (https://www.ebi.ac.uk/interpro/) (accessed on 22 May 2024). The first (Motif 1) and the second motifs (Motif 2) discovered by MEME (Figure 2b) were similar to the conserved SANT/myb protein domains predicted by InterPro (e.g., Pfam PF00249). Multiple sequence alignment by DNAMAN (Figure 2c) also exhibited the large overlaps of the R2R3-MYB domains discovered by MEME and Interpro. These results showed *SsMYB106*’s R2R3-MYB characteristics.

The *Agrobacterium*-mediated transient expression system co-expressing YFP and NLS-mCherry fusion proteins in *N. benthamiana* lower epidermal cells was conducted to determine the subcellular localization of *SsMYB106* (Figure 2d). The yellow fluorescence signals of control (pC131-free-YFP) were distributed in tobacco cell membrane, cytoplasm, and nucleus. The yellow fluorescence signals of pC131-*SsMYB106*-YFP fusion protein were in the nucleus and were completely merged with the red fluorescence signals of nuclear localization marker (pBin-NLS-mCherry), indicating that the pC131-*SsMYB106*-YFP fusion protein was exclusively present in the nucleus. These results demonstrate that *SsMYB106* was localized to the nucleus, consistent with its role as a TF.

The tissue-specific gene expression pattern analysis (Figure 2e) showed that *SsMYB10*6 were ubiquitously expressed in the root, stem, leaf, flower, and fruit, of which the stem and root were highly expressed organs. The *SsMYB106* transcriptomic absolute expressions of stem and root were 29.70-fold and 7.68-fold higher than other tissues. These results verified that the stem of *S. suberectus* was in a prominent position in the *SsMYB106* ubiquitous expressions.

The putative promoter *cis*-regulatory elements (CREs) analysis of *SsMYB106* identified ten light-response elements (four BOX4 (ATTAAT), two G-BOX (TACGTG), two GATA-motif (AAGATAAGATT), and two TCT-motif (TCTTAC)) (Table S1), suggesting that *SsMYB106* might be light responsiveness. To verify this, *SsMYB106* gene expressions under different light treatments were analyzed (Figure 2f,g). The RNA-seq data measured at absolute expression levels (Figure 2f) and quantitative reverse transcription polymerase chain reaction (RT-qPCR) data measured at relative expression levels (Figure 2g) exhibited similar change patterns: *SsMYB106* expressions were first irregular and in the long run tended to be decreased as the light intensity decreased. These results indicated that *SsMYB106* expression was regulated by light.

### 2.3. *SsMYB106* Was Closely Related to the Light-Intensity-Induced Changes of Flavonoids Contents and Pathway Genes Expressions in S. suberectus

In the promoter of MYB targeted genes, the MYB binding site is known for being bound by MYBs and responding to drought, light, flavonoid biosynthesis regulation, etc. [20]; the MYB recognition site can also be recognized and bound by MYBs [10]. Table S2 shows the promoter regions of 18 flavonoid biosynthetic genes in *S. suberectus*, except six genes (*SsPAL2*, *SsCHS*, *SsDFR2*, *SsLAR1*, *SsANR1*, and *SsANR2*). The remaining 12 genes (*SsPAL1*, *SsC4H1*, *SsC4H2*, *Ss4CL1*, *Ss4CL2*, *SsCHR*, *SsCHI*, *SsF3H*, *Ss3′H*, *SsDFR1*, *SsLAR2*, and *SsANS*) contained five types of MYB binding sites: (1) MYB binding site (CAACAG); (2) MYB binding site involved in flavonoid biosynthetic genes regulation (MBSI, aaaAaaC(G/C)GTTA); (3) MYB binding site involved in light responsiveness (MRE, AACCTAA); (4) MYB binding site involved in drought-inducibility (MBS, CAACTG); and (5) MYBHv1 binding site (CCAAT-box, CAACGG) (MYBHv1, a positive drought-tolerance regulator). Moreover, only two genes (*SsDFR1* and *SsANS*) contained the MYB recognition site (CCGTTG). These results demonstrate that most of the studied flavonoid biosynthetic genes were potentially MYB-regulated.

**Figure 3:**
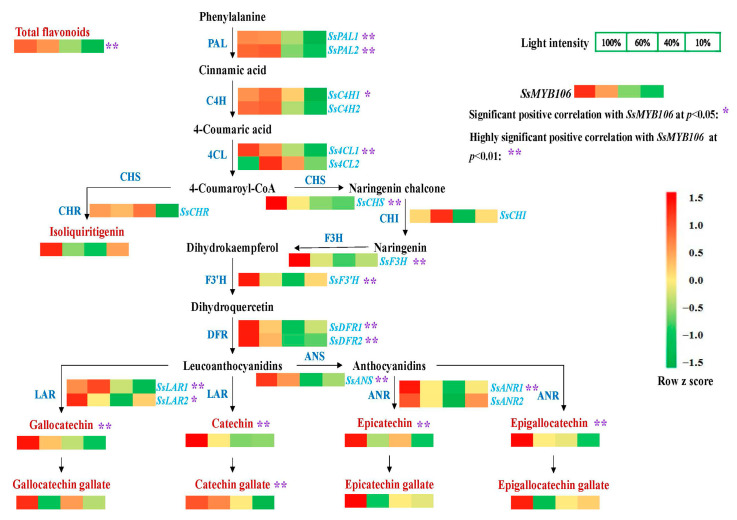
*SsMYB106* correlations with flavonoids and biosynthetic genes in light treatments. When two-year-old cultivated *S. suberectus* was treated with four attenuated sunlight intensities (100%, 60%, 40%, and 10%) for 60 days, fresh stem samples were collected, flavonoids contents were determined, and *SsMYB106* gene and 18 flavonoid biosynthetic genes expressions were quantified (RT-qPCR). Then, the Spearman’s rank correlations of *SsMYB106* gene expressions with flavonoids contents and with the 18 flavonoid biosynthetic genes were computed. Significant positive correlation at *p* < 0.05 is marked with a single asterisk (*), and highly significant positive correlation at *p* < 0.01 is marked with a double-asterisk (**). Abbreviations: PAL, phenylalanine ammonia-lyase; C4H, cinnamate-4-hydroxylase; 4CL, 4-coumaroyl-CoA ligase; CHS, chalcone synthase; CHR, chalcone reductase; CHI, chalcone isomerase; F3H, flavanone 3-hydroxylase; F3′H, flavonoid 3′-hydroxylase; DFR, dihydroflavonol-4-reductase; LAR, leocoanthocyanidin reductase; ANS, anthocyanidin synthase; ANR, anthocyanidin reductase.

To further elucidate the regulatory roles of *SsMYB106*, the light-intensity-induced change patterns of flavonoids (total flavonoids, eight catechins, and isoliquiritigenin) contents, the 18 biosynthetic pathway structural genes expressions, and their Spearman’s rank correlations with *SsMYB106* expressions in *S. suberectus* were analyzed. As shown in Figure 3, when treated with attenuated light intensity (100%, 60%, 40%, and 10%) for 60 days, flavonoids and the biosynthetic genes were varied with light intensity changes. Although their changed patterns were not always the same as *SsMYB106*, Spearman’s rank correlation analysis (Table S3, Figure 3) showed that, apart from isoliquiritigenin and three gallated catechins (epicatechin gallate, gallocatechin gallate, and epigallocatechin gallate), total flavonoids contents and the other five catechins (catechin, epicatechin, gallocatechin, epigallocatechin, and catechin gallate) were highly significantly correlated with *SsMYB106* expressions. Spearman’s rank correlation analysis (Table S4, Figure 3) also showed that, apart from *SsC4H2*, *Ss4CL2*, *SsCHR*, *SsCHI*, and *SsANR2*, the remaining 13 flavonoid biosynthetic genes expressions were all positively correlated with *SsMYB106* expressions, either significantly or highly significantly. These results demonstrate *SsMYB106*’s close relationship to the light-intensity-induced changes of flavonoids contents and their pathway genes expressions in *S. suberectus*.

### 2.4. *SsMYB106* Transient Overexpression Enhanced Flavonoids Accumulation, Increased All but One Pathway Genes Expressions in S. suberectus

**Figure 4:**
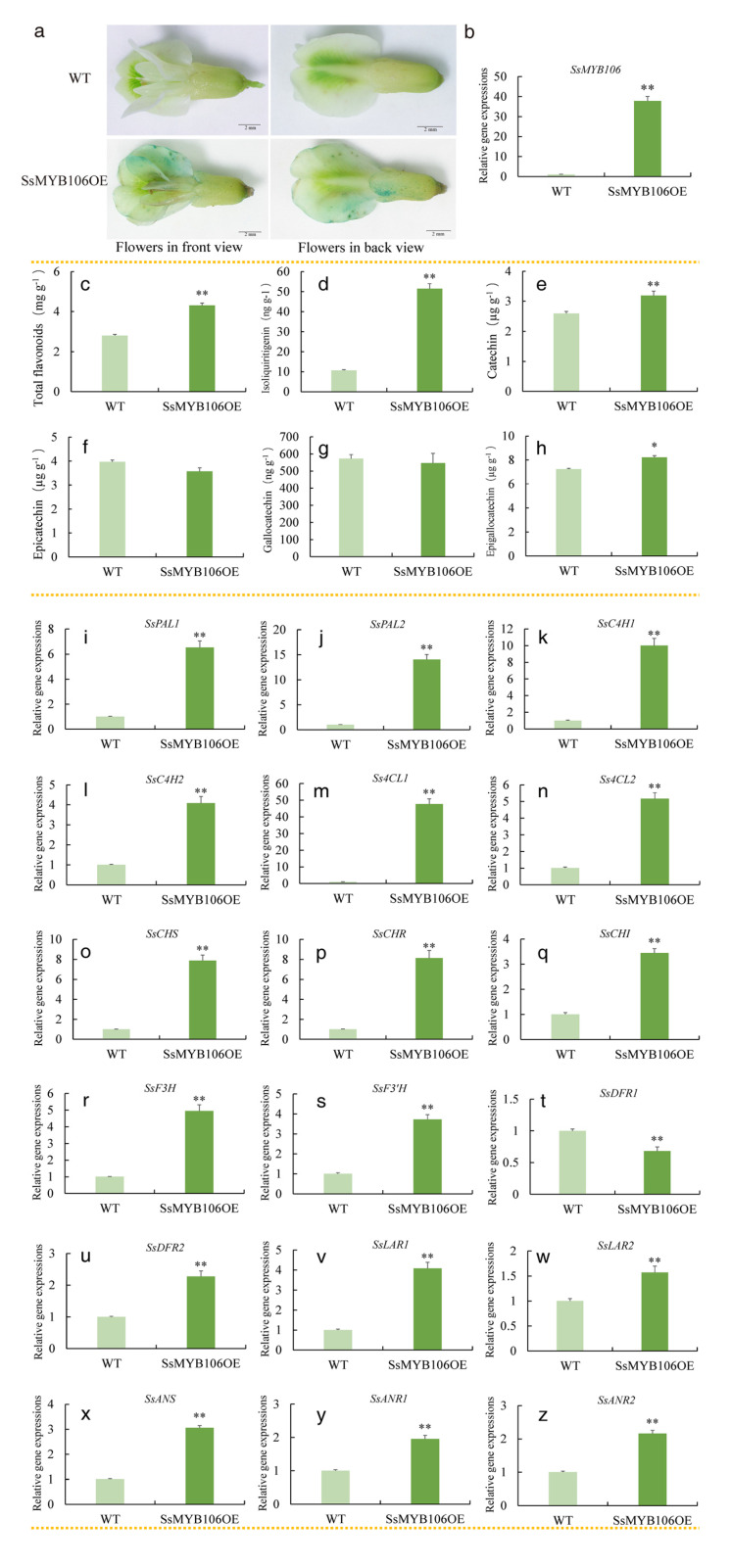
*SsMYB106* transient overexpression in *S. suberectus* flowers. (**a**) Gus staining of transient transformed pBI121-*SsMYB106*-GUS in *S. suberectus* flowers. *SsMYB106*OE, *SsMYB106*-overexpressing lines; WT, wild type. (**b**) *SsMYB106* gene expressions in WT and *SsMYB106*OE. (**c**–**h**) Flavonoids contents in WT and *SsMYB106*OE, including total flavonoids (**c**) and five specific flavonoid compounds (**d**–**h**). DW, dry weight. (**i**–**z**) Flavonoid biosynthetic genes expressions in WT and *SsMYB106*OE, 18 genes in total. Gene abbreviations are the same as in Figure 3. *, Significant positive correlation at *p* < 0.05. **, Highly significant positive correlation at *p* < 0.01.

As Figure 2e reveals, *SsMYB106* was expressed ubiquitously; transient overexpression of *SsMYB106* was attempted in stems, leaves, and flowers, but only succeeded in flowers. In *S. suberectus* blooming flowers, the constructed pBI121-*SsMYB106*-GUS recombinant vector was transiently overexpressed (Figure 4a). GUS signals (blue precipitates) were observed in flowers infiltrated with *Agrobacterium* carrying the constructed vector and expressing *SsMYB106*-GUS fusion protein (*SsMYB106*OE). GUS signals were not detected in wild type (WT). These results demonstrate that the *SsMYB106* gene was transiently overexpressed successfully.

*SsMYB106* expression was highly significantly increased by 37.78 folds in *SsMYB106*OE (Figure 4b). Total flavonoids, isoliquiritigenin, catechin, and epigallocatechin were highly significantly increased by 1.54-, 4.83-, 1.23-, 1.14-fold, respectively, as shown in Figure 4c–e,h. Epicatechin and gallocatechin were not significantly changed, as shown in Figure 4f,g. *SsDFR1* expression was significantly decreased by 0.32% in *SsMYB106*OE (Figure 4t). The other 17 flavonoid biosynthetic structural gene expressions were highly significantly increased in *SsMYB106*OEs, as shown in Figure 4i–s,u–z. Specifically, the increases of *SsPAL1*, *SsPAL2*, *SsC4H1*, *SsC4H2*, *Ss4CL1*, *Ss4CL2*, *SsCHS, SsCHR*, *SsCHI*, *SsF3H*, *SsF3′H*, *SsDFR2*, *SsLAR1*, *SsLAR2*, *SsANS*, *SsANR1*, and *SsANR2* were 6.54-, 14.04-, 10.01-, 4.08-, 47.73-, 5.17-, 7.88-, 8.14-, 3.45-, 5.00-, 3.72-, 2.27-, 4.08-, 1.57-, 3.06-, 1.96-, and 2.26-fold, respectively.

### 2.5. *SsMYB106* Stable Overexpression Promoted Flavonoids Accumulation, Activated the Majority of Pathway Genes Expressions in N. benthamiana

**Figure 5:**
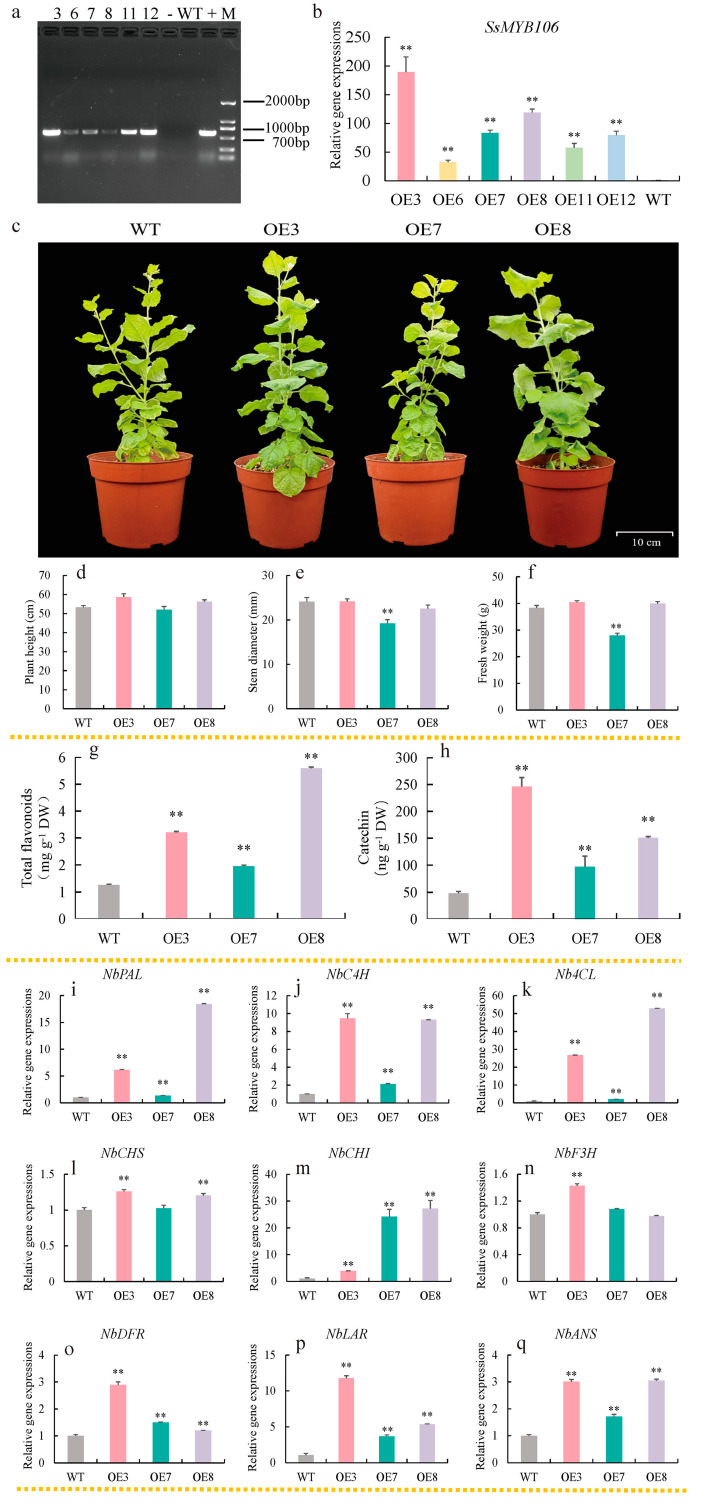
*SsMYB106* stable overexpression in *N. benthamiana*. (**a**) *SsMYB106* transgenic tobacco identification. Numbers ‘3, 6, 7, 8, 11, 12′, six *SsMYB106*-overexpressing lines (OE3, OE6, OE7, OE8, OE11, and OE12); -, negative control (containing empty vector pC131-YFP); WT, wild type; +, positive control (containing recombinant plasmid vector pC131-*SsMYB106*-YFP); M, DNA Marker-F, containing a mix of 6 individual DNA fragments (in base pairs): 200, 400, 700, 1000, 1500, 2000 bp. (**b**) *SsMYB106* gene expression in WT and six *SsMYB106*-overexpressing lines. (**c**–**q**) Comparisons between WT and the top three *SsMYB106*-overexpressing transgenic tobacco lines, including morphology (**c**), growth indices (**d**–**f**), flavonoids contents (**g**,**h**), and nine flavonoid biosynthetic pathway gene expressions (**i**–**q**). DW, dry weight. Gene abbreviations are the same as in Figure 3. **, Highly significant positive correlation at *p* < 0.01.

For stable genetic transformation, *Agrobacterium* carrying targeted vectors were transformed into *N. benthamiana* by the leaf disc method and the T_1_ generation lines were identified by PCR amplification, gel electrophoresis, and Sanger sequence. Of the 12 T1 transgenic lines, six *SsMYB106*-overexpressing lines (OE3, OE6, OE7, OE8, OE11, and OE12) were verified (Figure 5a). The *SsMYB106* relative expression levels in OE3, OE6, OE7, OE8, OE11, and OE12, as quantified by RT-qPCR, were 189.25-, 32.23-, 83.06-, 119.21-, 57.52-, and 79.78-fold higher than in WT, respectively (Figure 5b). The top three *SsMYB106*-overexpressing lines (OE3, OE8, and OE7) were chosen for further analysis.

In terms of phenotype, no differences were observed in the growth period, flowering time, and some morphological traits (leaf color, flower color, stem color, etc.) between three *SsMYB106*-overexpressing lines and WT (Figure 5c). The growth indices in *SsMYB106*-overexpressing OE3 and OE8 lines, such as plant height, stem diameter, and fresh weight, as shown in Figure 5d–f, were not significantly different to WT. But growth indices were partially inhibited in OE7 lines. As shown in Figure 5e,f, the stem diameter and fresh weight were highly significant decreased by 26.88% and 20%, respectively.

Contents of total flavonoids in the three *SsMYB106*-overexpressing lines (OE3, OE7, and OE8), as shown in Figure 5g, were highly significantly increased by 2.54-, 1.54-, and 4.42-fold, respectively, while contents of catechin in the three *SsMYB106*-overexpressing lines, as shown in Figure 5h, were highly significantly increased by 5.12-, 2.02-, and 3.14-fold, respectively.

Regarding the flavonoid biosynthetic gene expressions, all examined genes in the three *SsMYB106*-overexpressing lines were highly upregulated compared to WT, except for *NbCHS* in OE7 and *NbF3H* in OE7 and OE8, which remained unchanged. The specific fold changes in gene expressions were as follows: *NbPAL*, 6.15-, 1.34-, 18.42-fold (Figure 5i); *NbC4H*, 9.47-, 2.12-, 9.30-fold (Figure 5j); *Nb4CL*, 26.72-, 2.08-, 52.90-fold (Figure 5k); *NbCHS*, 1.26-, 1.02-, 1.21-fold (Figure 5l); *NbCHI*, 3.96-, 24.23-, 27.21-fold (Figure 5m); *NbF3H*, 1.43-, 1.08-, 0.97-fold (Figure 5n); *NbDFR*, 2.89-, 1.51-, 1.20-fold (Figure 5o); *NbLAR*, 11.78-, 3.66-, 5.34-fold (Figure 5p); and *NbANS*, 3.01-, 1.71-, 3.05-fold (Figure 5q).

These results demonstrate that *SsMYB106* stable overexpression produced two phenotypes, enhanced total flavonoids and catechin contents, and activated (or at least unchanged) all the flavonoid biosynthetic pathway genes expressions in transgenic *N. benthamiana*.

## 3. Discussion

### 3.1. *SsMYB106* Is Light-Regulated and Involved in Controlling the Light-Intensity-Induced Responses of S. suberectus

Flavonoids are the main medicinal components of *S. suberectus*, and our previous study found that flavonoids accumulations in *S. suberectus* were varied to adapt to the light environment [8]. To elaborate the molecular mechanisms behind the *S. suberectus’* responses to light, WGCNA was performed using the light-treated flavonoids content data and transcriptional RNA-seq data (Figure 1). Through WGCNA, MYB was screened as the top TF associating with light-intensity-induced flavonoid content changes in *S. suberectus*, and one *MYB* gene, namely, *SsMYB106*, was chosen for further analysis. Through analyzation of phylogeny (Figure 2a), conserved domains (Figure 2b,c), and protein subcellular localization (Figure 2d), it was discovered that the *SsMYB106* protein was closely related to its homologs of wild soybean and domesticated soybean, and it was an R2R3-MYB, exclusively localized to the nucleus. Through tissue-specific gene expression pattern (Figure 2e), it was found that the *SsMYB106* gene was ubiquitously expressed in all tissues but strongly expressed in the stem, which was in concert with the fact that the stem was also rich in flavonoids, especially catechins [8]. Through the presence of ten light-response elements in the *SsMYB106* promoter region (Table S1) and the light-intensity-induced absolute (Figure 2f) and relative (Figure 2g) *SsMYB106* expression pattern, it was verified that the *SsMYB106* gene was most likely in the light regulation network. Taken together, these results confirm that *SsMYB106* was characterized as a light-regulated R2R3-MYB TF in *S. suberectus*.

In addition to the aforementioned WGCNA, tissue-specific expression, and light-regulated expression, proof that *SsMYB106* gene functioned in controlling the light-intensity-induced responses of *S. suberectus* came from the following evidence: First, CREs analysis in *S. suberectus* revealed that 12 of the 18 flavonoid biosynthetic genes contained the MYB binding site and two genes contained the MYB recognition site (Table S2), providing the basis of these genes for being potentially MYB-regulated. Second, Spearman’s rank correlation analysis demonstrated that total flavonoids, 5 catechins, and 13 of the 18 flavonoid biosynthetic genes were significantly positively correlated with *SsMYB106* expressions (Tables S3 and S4), exhibiting the strong close relationship between flavonoid biosynthesis and *SsMYB106* expressions. Third, the overexpression of *SsMYB106*, both transiently in *S. suberectus* (Figure 4) and stably in *N. benthamiana* (Figure 5), affected flavonoids contents and nearly the whole flavonoid biosynthetic structural genes expressions, suggesting that *SsMYB106* was in the upstream regulation position of these biosynthetic pathway genes. However, direct evidence that *SsMYB106* protein interacted with the targeted genes (such as upstream light/hormone/stress signaling genes, genes in MBW complex/other interacting TFs, and downstream flavonoid biosynthetic pathway genes) should be experimentally validated in future studies.

### 3.2. *SsMYB106* Has Multifaceted Roles, Primarily Promotes Flavonoids Accumulation

#### 3.2.1. First, *SsMYB106* Overexpression Enhanced Flavonoids Accumulation

*S. suberectus* is an ornamental horticultural, medicinal, and edible plant in China. Flavonoids, especially catechins, endow *S. suberectus* with clinical and nutritional values. The present study experimentally revealed that both the *SsMYB106* transient overexpression in *S. suberectus* and the *SsMYB106* stable overexpression in *N. benthaminana* resulted in highly significantly increased total flavonoids contents (Figure 4 and Figure 5). The enhanced accumulation of total flavonoids was closely associated with the highly significantly increased genes expressions in the flavonoid biosynthetic pathway (Figure 4 and Figure 5). These increases brought about two outcomes: One was the obvious increase in flavonoid compounds, which was detected in the present study, such as flavanols (catechin, epicatechin, gallocatechin, and epigallocatechin) and chalcones (isoliquiritigenin); the other was the unobserved increase in intermediate flavonoids. For example, the increased expression of *SsCHI* would directly enhance its product—naringenin. Naringenin can produce dihydroflavonols (dihydrokaempferol and dihydroquercetin) through F3H and F3′H, flavone (e.g., luteolin, apigenin, and 3′,4′,7-trihydroxyflavone) through flavone synthase (FNS), and isoflavone (e.g., daidzin, genistein, formononetin, and calycosin) through isoflavone synthase (IFS) [7]. All was favorable for the total flavonoids accumulation.

#### 3.2.2. Second, *SsMYB106* Overexpression Preferably Directed Metabolic Flux into Catechin Biosynthesis

In plants, anthocyanins usually exhibit red, orange, purple, and blue colors and are influenced by structure modifications, light-yellow or even colorless co-pigments (e.g., flavonol and flavones), pH, metal ions (e.g., Al^3+^, Fe^3+^, and Mg^2+^), light, temperature, sugar (sucrose, glucose, and fructose), oxygen, etc. [21,22,23]. And there is broad agreement that in the anthocyanin biosynthetic pathway, *CHS*, *CHI, F3H*, *F3′H*, *F3′5′H* (encoding flavonoid *3*′*5*′-hydroxylase, not in *S. suberectus*’ genome), *DFR*, *ANS*, and *UFGT* (encoding UDP-glucose flavonoid 3-O-glucosyl transferase, functioned in anthocyanidins 3-glucosylation) are critical structural genes whose expressions affect color development and alter color phenotypes [6,24,25,26,27,28]. Additionally, MYB TFs and MBW complex were also proved to transcriptionally regulate (activate or suppress) these structural genes and, thus, had a great impact on plant color [9,29,30,31,32,33,34]. However, in the present study, even though most of the associated crucial genes were elevated when *SsMYB106* was overexpressed, no color variations (no increased/decreased anthocyanin pigmentation and other color modifications) were observed in the flowers of transiently overexpressed *S. subrectus* and in the flowers, leaves, and stems of stably overexpressed *N. benthaminana* (Figure 4 and Figure 5). This phenomenon indicates that anthocyanin biosynthesis is repressed to prevent pigmentation and maintain normal colors in *SsMYB106*-overexpressing plants, although the actual anthocyanins contents were not determined in this study. Combined with the results that *SsMYB106* overexpression increased contents of flavonoids, especially catechins (catechin, highly significantly increased in *S. suberectus* and in *N. benthamiana*; epigallocatechin, significantly increased in *S. suberectus*) (Figure 4 and Figure 5), it was concluded that *SsMYB106* overexpression led to significantly enhanced catechins but reduced anthocyanins, indicating that *SsMYB106* preferably directed metabolic flux into catechin biosynthesis and avoided unnecessary anthocyanins production. This will be discussed for *S. suberectus* and in *N. benthamiana*, respectively.

##### In *S. suberectus*

To clarify, *SsDFR1* and *SsDFR2* function differently in the flavonoid biosynthetic pathway. This could be deduced from four aspects. First, in addition to the DNA sequence difference, the putative CREs in their promoter regions was different: *SsDFR1* contained one MYB recognition site and three MYB binding sites while *SsDFR2* contained none of them (Table S2), indicating that they may be differently regulated by MYB-TFs. Second, SsDFRs enzymes have two substrates in *S. suberectus*: dihydrokaempferol and dihydroquercetin. SsDFRs can reduce dihydrokaempferol into leucopelargonidin or reduce dihydroquercetin into leucocyanidin. Despite not yet being experimentally validated, substrate specificity probably exists in *SsDFR1* and SsDFR2, implying that they may switch to different metabolic pathways (flavanols/flavonols/anthocyanins) or affect the proportion of pelargonidin/cyanidin-based anthocyanins [30]. Third, most importantly, *SsDFR1* and *SsDFR2* exhibited distinct gene expression pattern when *SsMYB106* was transiently overexpressed in *S. suberectus* flowers: *SsDFR1* was remarkably decreased while *SsDFR2* was remarkably increased, further implying that they may play different roles in flavonoid biosynthesis. Fourth, our previous study revealed that *SsDFR1* and *SsDFR2* displayed differential tissue-specific expression patterns: *SsDFR1*(*DFR*_Chr5.130) was expressed in the root, stem, leaf, and flower, and highly expressed in the flower, in which anthocyanins were richly deposited; *SsDFR2* (*DFR*_Chr5.129) was expressed in the root, stem, leaf, flower, and fruit, and highly expressed in the stem, in which catechins were abundantly accumulated [7]. Taken together, it was concluded that *SsDFR1* and *SsDFR2* functioned differently: *SsDFR1* was mainly involved in anthocyanin biosynthesis, and *SsDFR2* was mainly involved in catechin biosynthesis.

On this basis, the results that *SsMYB106* transient overexpression in *S. suberectus* led to enhanced catechins and repressed anthocyanins were reasonable: In the catechins branch pathway, all related genes—*SsDFR2*, *SsLAR1*, *SsLAR2*, *SsANS*, *SsANR1*, and *SsANR2*—were highly significantly increased, thereby enhancing catechin biosynthesis. In the anthocyanins branch pathway, *SsDFR1* played crucial roles in counteracting promotion effects of all other elevated genes (*SsCHS*, *SsCHI*, *SsF3H*, *SsF3′H*, and *SsANS*), thus inhibiting anthocyanins production. But other factors, such as genes (e.g., *FLS*, *ANR*) that negatively regulate anthocyanin biosynthesis [35,36]; genes involved in anthocyanin modification [37,38], transport [39], and degradation [40,41]; and genes in repression modules [42] might be involved in repressing anthocyanin.

Another explanation for the repressed anthocyanin biosynthesis was the competition between *SsDFR1* and the flavonol synthase encoding gene *SsFLS*. As was proved in other plants [43,44,45,46], in *S. subrectus* it was most likely that the enzymes of *SsFLS* and SsDFR competed with the common substrate—dihydrokaempferol—to produce flavonol (e.g., kaempferol and quercetin) and leucoanthocyanidin (leucopelargonidin and leucocyanidin), respectively, and their encoding genes mutually inhibited the expressions of each other. Therefore, when *SsMYB106* was overexpressed, the *SsDFR1* expression decreases promoted the *SsFLS* expression increases, and thereby promoting non-colored flavonol biosynthesis and inhibiting colored anthocyanin biosynthesis.

Therefore, to conclude, in the regulation of flavonoid biosynthesis in *S. suberectus*, *SsMYB106* acted as an activator by promoting *SsDFR2* and all catechin-related genes expressions to enhance catechin biosynthesis. *SsMYB106* might also act as a repressor by inhibiting *SsDFR1* expression or interacting with other factors to suppress anthocyanin biosynthesis.

##### In *N. benthamiana*

The enhancement of catechins in *N. benthamiana* was plausible because all related genes, especially the two direct catechin biosynthetic genes, *NbDFR* and *NbLAR*, were highly significantly upregulated by *SsMYB106* overexpression (Figure 5).

In contrast, the regulation of anthocyanins by *SsMYB106* in *N. benthamiana* was relatively complicated. Normally, the effects of MYB TFs introduction on the regulation of anthocyanins in *N. benthaminana* depend on MYB type, origin, controlled promoter, co-expressed proteins (usually bHLHs), etc. For instance, a previous study introducing *Rosea1* (*ROS1*, an R2R3-MYB TF from *Antirrhinum majus* L.) under the control of *Floral Binding Protein 1* (*FBP1*, a flower-specific promoter from *Petunia* × *atkinsiana* D.Don ex Loudon (i.e., *Petunia hybrid* (Hook.) E. Vilm.)) into *N. benthamiana* produced growth-normal plants with purple flowers, while the introduction of *ROS1* under the control of CaMV *35S* promoter generated stunted plants with deep reddish-brown plants (too many anthocyanins accumulated in all plant parts) or phenotypically normal plants with white flowers; and the color modification in transgenic *N. benthamiana* was attributed to delphinidin accumulation resulting from stimulated expressions of *NbCHS*, *NbF3H*, *NbDFR*, and *NbANS* [47]. Further research demonstrated that the natural anthocyanins in *N. benthaminana* were pelargonidin-based (produced by F3H) and delphinidin-based (produced by F*3′5′*H), and none were cyanidin-based (produced by F*3′*H) anthocyanins; and for anthocyanin biosynthesis, four genes (*CHS*, *DFR*, *ANS*, and *GST* (encoding glutathione S-transferase, functioned in transporting and sequestrating anthocyanins to vacuole)) were absolutely required [48]. In the present study, *SsMYB106*, an R2R3-MYB TF from *S. suberectus*, was introduced into *N. benthamiana* under the control of the CaMV *35S* promoter. The overexpression of *SsMYB106* in *N. benthamiana* produced transgenic normal-growing plants and inhibited-growth plants (slim stem and less biomass accumulation), both bearing white flowers (similar to WT). The corresponding pathway genes, namely, *NbPAL*, *NbC4H*, *Nb4CL*, *NbCHS*, *NbCHI*, *NbF3H*, *NbDFR*, *NbLAR*, and *NbANS*, were all activated (or at least unchanged) in transgenic plants (Figure 5). Thus, the effects of the introduction of *SsMYB106* TF into *N. benthamiana* were unlike the reported TFs. The reason that the stimulated anthocyanins pathway genes did not lead to color variations in *N. benthamiana* was uncertain, whether because *SsMYB106* interacted with the endogenous bHLH and other TFs (e.g., MYBs) [49], or because *SsMYB106* also influenced the later genes (not studied presently) in anthocyanin biosynthesis (e.g., *UFGT*) and transport (e.g., *GST*), or other reasons (e.g., small RNAs) [42]. The exact mechanisms need to be explored experimentally in future.

In sum, in *N. benthamiana*, *SsMYB106* activates catechin biosynthesis by increasing catechin-related genes, and suppresses anthocyanin biosynthesis by currently undetermined mechanisms.

#### 3.2.3. Third, *SsMYB106* Overexpression Might Inhibit Lignin Biosynthesis

In *SsMYB106* stably overexpressed *N. benthamiana*, OE7 lines exhibited remarkably slim stems and reduced biomass accumulation (Figure 5), which were two typical traits among the multiple phenotypic characteristics induced by the suppression of lignin biosynthesis [50,51]. Hence, it was speculated that these phenotypic defects in OE7 lines might be the reduced lignin biosynthesis caused by *SsMYB106* overexpression. In the phenylpropanoid pathway, a major branch other than flavonoid biosynthesis is lignin biosynthesis [52]. PAL, C4H, and 4CL were also functioned in lignin biosynthesis [53]. The C4H product—4-coumaric acid, and the 4CL product—4-coumarate CoA, can channel metabolic flux into monolignol biosynthesis by 4-coumarate 3-hydroxylase (C3H) and hydroxycinnamoyl-CoA shikimate/quinate hydroxycinnamoyl transferase (HCT), respectively [54]. Moreover, CHS competes with HCT for substrate 4-coumarate CoA and determines the metabolic flux entering into flavonoid biosynthesis or lignin biosynthesis [55]. In addition, besides lignins and flavonoids, 4-coumarate CoA also mediates the synthesis of lignans, coumarins, phenolic glycosides, phenylpropanoid esters, etc. [56]. Taken together, because of the competitive relationship between lignin and flavonoid biosynthesis and some involved intermediate metabolites, the lignin biosynthesis might be inhibited indirectly by the promotion of flavonoid biosynthesis in *SsMYB106*-overexpressing OE7 lines.

However, the OE3 and OE8 lines with enhanced expressions of flavonoid-associated genes exhibited normal growth and did not show any phenotypic differences to WT, suggesting the good homeostasis between elevated flavonoid biosynthesis and other plant developmental processes when *SsMYB106* was overexpressed [57,58]. One possible reason was that *SsMYB106* could be redundantly controlled by other *MYB* gene family members or by the MBW complex [19]. For example, recently, we demonstrated that *SsMYB158*, another R2R3-MYB in *S. subrectus*, also functioned in enhancing flavonoids, especially catechins, but the *SsMYB158*-overexpressing *N. benthamiana* plants remained normal-growing and exhibited no phenotypic defects [59]. However, the co-operation mechanisms of *SsMYB106* with other *MYBs* also need to be experimentally validated.

### 3.3. Proposed Model and Future Work

#### 3.3.1. Proposed Model

**Figure 6:**
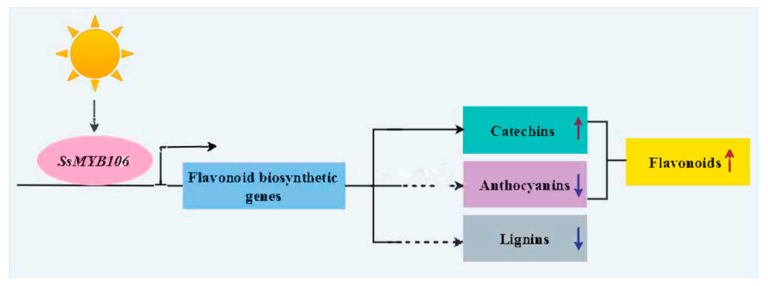
*SsMYB106* gene function model. *SsMYB106* was regulated by light and activated/repressed targeted flavonoid biosynthetic genes. *SsMYB106* transient overexpression in *S. suberectus* flowers and stable overexpression in *N. benthamiana* ultimately promoted flavonoids accumulation and activated catechin biosynthesis. *SsMYB106* overexpression in the two plants altered expressions of all anthocyanin-related genes with no color variations, implying that anthocyanin biosynthesis was repressed to avoid unnecessary anthocyanins production. *SsMYB106* overexpression in *N. benthamiana* produced phenotypically normal plants and inhibited-growth plants; the latter might be caused by inhibited lignin biosynthesis. The up arrow symbol indicates promotion/activation, and the down arrow symbol indicates inhibition/repression. The solid line indicates solid evidence in this study, while the dashed line indicates indirect evidence and needs to be further experimentally validated in the future.

A working model was proposed to illustrate *SsMYB106* gene functions (Figure 6). *SsMYB106* was in the upstream of the light-regulated flavonoid biosynthetic network, responding to light, and participated in controlling downstream target genes. *SsMYB106* overexpression in *S. suberectus* and in *N. benthamiana* upregulated the majority of flavonoid pathway genes, promoted flavonoids (especially catechins) biosynthesis, and suppressed anthocyanin biosynthesis. Part of the *SsMYB106*-overexpressing tobacco lines exhibited phenotypical defect traits relating to reduced lignins, and implied that *SsMYB106* overexpression might inhibit lignin biosynthesis.

#### 3.3.2. Future Work

To validate and extend the present findings, at least four things should be performed in future research: The first is to determine the contents of anthocyanin and lignin, which will be the direct evidence used to confirm the phenotypic differences caused by *SsMBY106* gene overexpression. The second is to study the gene functions of *SsDFR1* and *SsDFR2*, which will experimentally discriminate their exact roles in flavonoid biosynthesis. The third is to explore protein–DNA (e.g., yeast one-hybrid (Y1H) and electrophoretic mobility shift assay (EMSA)), protein–protein (e.g., yeast two-hybrid (Y2H), and bimolecular fluorescence complementation (BiFC)) interactions, which will identify the direct target genes (promoters)/proteins interacting with the *SsMYB106* protein. The fourth is to knock out (e.g., CRISPR-Cas9) or knock down (e.g., RNAi) the *SsMYB106* gene or its redundantly controlled *MYB* gene family members or MYB complex members, which will be another solid piece of proof to elucidate *SsMYB106* gene functions.

## 4. Materials and Methods

### 4.1. Plant Material

**Figure 7:**
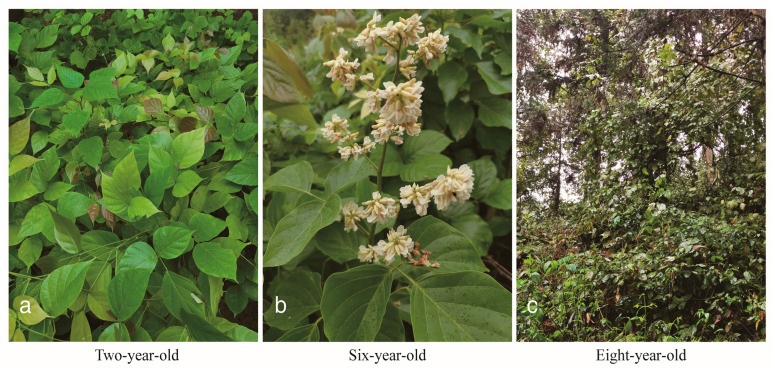
Plant material. Two- (**a**), six- (**b**), and eight-year-old (**c**) cultivated *S. suberectus* used for light treatments, *SsMYB106* transient expression, and *SsMYB106* tissue-specific gene expression, respectively.

*Spatholobus suberectus* Dunn was cultivated as described in our previous study [8]. As flowers and fruits can only be gathered from perennial growers, *S. suberectus* of different cultivating years were used in this study. Specifically, two-year-old (2019–2020) (Figure 7a), six-year-old (2017–2022) (Figure 7b), and eight-year-old (2010–2017) (Figure 7c) cultivated *S. suberectus* Dunn plants were used for light treatments, *SsMYB106* transient expression, and *SsMYB106* tissue-specific gene expression, respectively. In addition, *Nicotiana benthamiana* Domin was cultivated at 25 °C, 16/8 h (light/dark), and 4500 lux (light intensity) for *SsMYB106* subcellular localization and stable genetic transformation.

### 4.2. Light Treatments

Light treatments were performed as described in our previous study [8]. Briefly, four attenuated sunlight intensities (100%, 60%, 40%, and 10%) and three durations (30 d, 45 d, and 60 d) were applied to two-year-old cultivated *S. suberectus*. After each light treatment, fresh stem samples were collected for flavonoids determination and genes expression quantification.

### 4.3. Flavonoids Content Determination

Contents of flavonoids, including total flavonoids, eight catechins (catechin, epicatechin, gallocatechin, epigallocatechin, catechin gallate, epicatechin gallate, gallocatechin gallate, and epigallocatechin gallate), and isoliquiritigenin in *S. suberectus*, and contents of total flavonoids and catechin in *N. benthamiana* were determined as described in our previous study [8].

### 4.4. Transcriptomic Gene Expression

The RNA-seq data were used for absolute quantification of gene expressions. The RNA library construction and transcript quantification (transcripts per million reads, TPM) were performed as described in our previous study [8].

### 4.5. RT-qPCR

RT-qPCR data were used for relative quantification of gene expressions. For RT-qPCR, two assay kits, HiScript^®^ III RT SuperMix for qPCR (+gDNA wiper) (Vazyme Biotech Co., Ltd., Nanjing, China), and *PerfectStart*^®^ Green qPCR SuperMix (+Dye II) (TransGen Biotech Co., Ltd., Beijing, China), were used. Gene-specific primers of *SsMYB106* and 18 flavonoid biosynthetic genes (*SsPAL1*, *SsPAL2*, *SsC4H1*, *SsC4H2*, *Ss4CL1*, *Ss4CL2*, *SsCHS*, *SsCHR*, *SsCHI*, *SsF3H*, *SsF3′H*, *SsDFR1*, *SsDFR2*, *SsLAR1*, *SsLAR2*, *SsANS*, *SsANR1*, and *SsANR2*) in *S. suberectus* (Table S6), and nine flavonoid biosynthetic genes (*NbPAL*, *NbC4H*, *Nb4CL*, *NbCHS*, *NbCHI*, *NbF3H*, *NbDFR*, *NbLAR*, and *NbANS*) in *N. benthamiana* (Table S7), were designed by Primer Premier 5.0 (PREMIER Biosoft International, San Francisco, CA, USA). *Ss18S* and *NbEF1α* were used as internal reference genes in *S. suberectus* and in *N. benthamiana*, respectively. RT-qPCR was performed on Applied Biosystems QuantStudio™ 3 Real-time System (Thermo Fisher Scientific Inc., Waltham, MA, USA), and relative gene expression levels were computed by the 2^−ΔΔCT^ method [60].

### 4.6. WGCNA

The flavonoids content data (total flavonoids, eight catechins, and isoliquirigenin) and the whole RNA-seq absolute gene expression data in the abovementioned light treatments were used to build a weighted gene co-expression network using the WGCNA package (Version 1.47) [61]. The typical analysis steps were as follows: First, we built the clustering dendrogram tree based on topological overlap matrix (TOM) dissimilarity distance, and the parameters set were the following: Power = 13 (the appropriate soft threshold (β), chosen to fit scale-free topology model), Min.Module Size = 30 (a minimum number of 30 genes in each module), and Merge cut Height = 0.25 (corresponding to a correlation of 0.75). Second, we constructed a module-trait Pearson correlation heatmap to identify biologically meaningful modules. Third, we performed Gene Ontology (GO) enrichment analyses of the positively correlated gene modules to screen candidate genes regulating flavonoid biosynthesis in *S. suberectus*. In addition, the top ten WGCNA screened TFs were presented in a graph for clarity.

### 4.7. Phylogeny Tree

To construct the phylogenetic tree, the protein sequence of *SsMYB106* was used for Protein BLAST (Version 1.4.0) query via National Center for Biotechnology Information (NCBI) to retrieve homologous protein sequences. Altogether, 14 species listed below, including VuODO1, *Vigna umbellata,* XP_047158282.1; VaODO1, *Vigna angularis*, XP_052733598.1; VrMYB8, *Vigna radiata* var. *radiate*, XP_014509393.1; VuODORANT1, *Vigna unguiculata*, XP_027917244.1; GsODORANT1, *Glycine soja*, XP_028209493.1; GmMYBJ1, *Glycine max*, NP_001241087.1; CcODORANT1, *Cajanus cajan*, XP_020228788.1; ApODORANT1, *Abrus precatorius*, XP_027334263.1; MtODO1, *Medicago truncatula*, XP_013457650.1; LaODORANT1, *Lupinus angustifolius*, XP_019422294.1; PaODORANT1, *Prosopis alba*, XP_028762533.1; MdMYB54, *Malus pumila*, AUZ96353.1; DlMYB16, *Dimocarpus longan*, QCH41128.1; and PsMYB23, *Paeonia* × *suffruticosa*, QIG55706.1, along with *SsMYB106* (*Spatholobus suberectus*, XP_ TKY55375.1), were chosen to perform phylogenetic analysis using MEGA 11 (Molecular Evolutionary Genetics Analysis Version 11) software (https://megasoftware.net/) (accessed on 22 May 2024). Sequences were first aligned by the ClustalW algorithm and subsequently used for constructing a neighbor-joining tree using the bootstrap method with 1000 bootstraps. The 15 amino acid sequences for phylogenetic tree analysis are listed in Table S5.

### 4.8. Motif Discovery

Sequences for phylogenetic analysis were input into MEME [62] (Version 5.5.2) (https://meme-suite.org/meme/tools/meme) (accessed on 22 May 2024) to discover motifs. ZOOPS model (assumes zero or one occurrence per sequence of the motif) was used as the motif discovery algorithm. These sequences were also input into InterPro [63] (https://www.ebi.ac.uk/interpro/) (accessed on 22 May 2024) to search for conserved protein domains. For instance, InterPro Pfam entry—PF00249 (https://www.ebi.ac.uk/interpro/entry/pfam/PF00249/) (accessed on 22 May 2024), a characteristic Myb-like DNA-binding domain, is used for identifying MYB proteins. Multiple sequence alignment was constructed by DNAMAN (Version 6.0) to reveal the conserved domain sequences and compare results of MEME and InterPro.

### 4.9. Subcellular Localization

For subcellular localization, the pEASY^®^-Basic Seamless Cloning and Assembly Kit (TransGen Biotech Co., Ltd., Beijing, China) was used to insert the coding sequence (CDS) of *SsMYB106* into an overexpression vector pC131-YFP (carrying enhanced yellow fluorescent protein (EYFP) reporter gene) by In-Fusion cloning. First, primers were designed using the “double digestion linearized” method (via *EcoR*I and *Spe*I restriction sites) on the TransGen Biotech website (https://soft.transgen.com/twoh.php) (accessed on 2 March 2023). Primers used to construct fragment inserts with homology ends are listed in Table S7. Second, the linearized pC131-YFP vector was generated by the same restriction endonucleases digestion. Third, the fragment inserts with 15–25 bp overlapping ends and the linearized vector with homologous ends were seamlessly assembled. Fourth, the recombinant pC131-*SsMYB106*-YFP plasmid was propagated in *Escherichia coli* DH5α component cells (Vazyme Biotech Co., Ltd., Nanjing, China). In the end, when the In-Fusion cloning was performed, plasmids of the recombinant (pC131-*SsMYB106*-YFP), empty vector control (pC131-free-YFP), and nucleus marker (pBin-NLS-mCherry, a red fluorescent protein fused with nuclear localization signal (NLS) of the simian virus 40 (SV40) large T antigen to target red fluorescence to nuclei) were separately transformed into *Agrobacterium tumefaciens* (strain GV3101) through the liquid nitrogen freeze–thaw technique, and the transformed agrobacteria were incubated routinely. Before co-transformation, *Agrobacterium* containing control/recombinant plasmids were separately mixed with *Agrobacterium* containing nuclear marker plasmid. Then, the *Agrobacterium* mixture harboring targeted plasmids (pC131-free-YFP + pBin-NLS-mCherry, and pC131-*SsMYB106*-YFP + pBin-NLS-mCherry) were infiltrated into four-leaf-stage *N. benthamiana*. After being incubated for 3~5 days, the lower epidermal cells of transformed tobacco leaves were taken for fluorescence signals observation. Yellow (EYFP, excitation peak at 513 nm and emission peak at 527 nm) and red (mCherry, excitation peak at 587 nm and emission peak at 610 nm) fluorescence signals were visualized under a Leica TCS SP8 Confocal Microscope (Lecia Microsystems, Wetzlar, Germany; Danaher Life Sciences, Danaher Corporation, Washington, United States).

### 4.10. Gene Promoter CREs Analysis

PlantCARE [64,65] (https://bioinformatics.psb.ugent.be/webtools/plantcare/html/) (accessed on 10 June 2023) was used for CREs prediction. The promoter sequence (~2 kb upstream from the transcription start codon ATG) of *SsMYB106*, and 18 biosynthetic flavonoids pathway genes (*SsPAL1*, *SsPAL2*, *SsC4H1*, *SsC4H2*, *Ss4CL1*, *Ss4CL2*, *SsCHS*, *SsCHR*, *SsCHI*, *SsF3H*, *SsF3′H*, *SsDFR1*, *SsDFR2*, *SsLAR1*, *SsLAR2*, *SsANS*, *SsANR1*, and *SsANR2*) were input into the database and searched for light-responsive CREs and MYB-related CREs (MYB binding site and MYB recognition site), respectively.

### 4.11. *SsMYB106* Transient Overexpression in S. suberectus Flowers

Two steps were taken for *SsMYB106* gene transient overexpression in the blooming flowers of six-year-old cultivated *S. suberectus*. The first step was recombinant plasmid construction and *Agrobacterium*-mediated transient transformation. The recombinant plasmid pBI121-*SsMYB106*-GUS was constructed by cloning the CDS of *SsMYB106* into a pBI121 binary plasmid vector, which contains a GUS (β-Glucuronidase) reporter gene driven by cauliflower mosaic virus (CaMV) *35S* promoter and nopaline synthase (NOS) terminator, and a selectable marker gene *npt*II (neomycin phosphotransferase, conferring kanamycin resistance) driven by the NOS promoter and the NOS terminator. Primers used in vector construction are listed in Table S6. Then, the recombinant plasmid was introduced into *A. tumefaciens* strain GV3101 by the freeze–thaw method. The transformed *Agrobacterium* was cultured overnight at 28 °C in Luria–Bertani (LB) liquid medium containing 50 μg/mL kanamycin. Then *Agrobacterium* cells were collected, centrifuged, suspended to an optimal density, and infiltrated into blooming flowers of *S. suberectus*. The second step was the GUS histochemical staining performed with the GUS Staining Kit (Biosharp, Beijing Labgic Technology Co., Ltd., Beijing, China). The transient transformed flowers (*SsMYB106*OE) and wild type flowers (WT, control) were immersed in GUS staining solution and incubated in the dark for 16 h. Stained flowers were visualized using an Olympus SZX7 stereo microscope coupled to an Olympus DP27 microscope digital camera (Evident Corporation, Tokyo, Japan).

### 4.12. *SsMYB106* Stable Overexpression in N. benthamiana

For stable genetic transformation, the overexpression vector pC131-YFP carrying hygromycin- and kanamycin-resistant genes were used. *A. tumefaciens* GV3101 harboring targeted vectors (recombinant plasmid vector pC131-*SsMYB106*-YFP and empty control vector pC131-YFP) were transformed into *N. benthamiana* by the following leaf disc method: First, aseptic leaves of four-week-old tobacco plants were cut into pieces, soaked in *Agrobacterium* solution for 10 min, dried with sterile filter paper, cultured in co-cultivation medium, and incubated in the dark for 48 h. Second, the co-cultivated leaf discs were cultured in resistance screening medium and incubated under light until the sprouted young shoot appeared. Third, the resistant shoots were continuously cultured to form elongated shoots, roots, and, finally, the complete plantlet. Fourth, the positive transgenic tobacco plants were selected and validated. The Plant Direct PCR Kit (Vazyme Biotech Co., Ltd., Nanjing, China) was used to amplify target sequences. Primers specific for *SsMYB106* polymerase chain reaction (PCR) amplification are listed in Table S6. The PCR reaction products were assessed by agarose gel electrophoresis. DNA Marker F (200~2000 bp) (Songon Biotech, Shanghai, China) was used to read the electrophoretic bands. The targeted gel bands were then sequenced by Sanger sequencing for further validation. By this means, the T_0_ generation transgenic tobacco lines were validated. Later, the T_0_ generations were harvested to collect T_1_ seeds. Finally, the T_1_ seeds were sterilized and screened in hygromycin-resistant MS medium. Then, the T_1_ generations of transgenic tobacco lines were also identified by the conjunction of PCR amplification, agarose gel electrophoresis, and Sanger sequencing, and the confirmed positively overexpressed T1 lines were selected for further analysis.

### 4.13. Statistical Analysis

Statistical analyses were carried out as described in our previous study [8]. IBM^®^ SPSS^®^ Statistics 19 (IBM, New York, NY, USA) was used for analyzation. One-way analysis of variance (ANOVA) with Duncan’s post hoc test was performed to compare multiple groups’ means differences. The Spearman’s rank correlation significance test was used to interpret the relationship between two variables. The probability (*p*) value set for determining statistical significance was *p* < 0.05 as statistically significant and *p* < 0.01 as highly statistically significant.

## 5. Conclusions

In conclusion, WGCNA identified *MYB* genes as the potential regulators for light treatments. The phylogeny, sequence, localization, expression, and association depicted *SsMYB106* as a light-regulated R2R3-MYB participating in light-intensity-induced responses. *SsMYB106* transient and stable transformation promoted flavonoids accumulation and activated catechin biosynthesis. The altered expressions of anthocyanin-related genes and unaltered colors of *SsMYB106*-transformed plants implied that anthocyanin biosynthesis might be repressed to avoid unnecessary anthocyanins production. The differentiation of phenotypically normal and inhibited-growth in transformed tobacco indicated that lignin biosynthesis might be partially inhibited. As a member of the gene family and the MBW complex, the *SsMYB106* gene function also might be affected by family genes or by MBW partners. The deepened understanding of *SsMYB106* gene function would be beneficial for ultimately designing flavonoid biosynthesis.

## Data Availability

Data are contained within the article and Supplementary Materials.

## Supplementary Materials

The following supporting information can be downloaded at: https://www.mdpi.com/article/10.3390/ijms26115292/s1.
